# Supplementary material for: A retrospective analysis to estimate the healthcare resource utilization and cost associated with treatment-resistant depression in commercially insured US patients
Source: PLoS One. 2020 Sep 11;15(9):e0238843. doi: 10.1371/journal.pone.0238843 (PMC7485754; doi:10.1371/journal.pone.0238843)
Supplement: S1 Appendix — (DOCX) [file pone.0238843.s001.docx]

**S1 Appendix. High dimensional covariate selection approaches.**

*Description of methods*

Two high dimensional covariate selection (HD) approaches were used as sensitivity analyses to select potential new covariates, in addition to the pre-specified covariates for propensity score (PS) matching in the study.

The first HD approach (HD1) was to select 40 covariates from the 20 most frequent diagnoses and another 20 most frequent medications used during the baseline period, in addition to the pre-specified covariates. The second HD (HD2) approach was to use the Schlesselman score method to select the top 50 empirical covariates that were associated with a risk of high cost from three domains of data: diagnoses, medications, and treatment procedures during the baseline period. Here, the high cost group was defined as total cost above the 90th percentile.

Three covariate sets (ie, pre-specified, HD1, and HD2) were considered for the analysis. For each covariate set, three covariate balancing approaches were applied: 1) Mahalanobis distance Matching (MDM); 2) Propensity score matching (PSM); 3) Stratification by PS (PS_strata), 10 strata from 10 quantiles of PS score. Therefore, a total of 9 sets of analyses were performed.

The differences of least square means and their confidence intervals of total cost in treatment-resistant depression versus non–treatment-resistant depression were estimated through linear mixed effects model for repeated measurements on years 1 and 2.

*Results*

Additional covariates selected via HD1 and HD2 are presented in Table A and Table B, respectively. The number of patients included in each analysis is tabulated in Table C. Differences in least square means of total cost to payers (US$) estimate between treatment-resistant depression and non–treatment-resistance major depressive disorder for the nine sets of analyses are shown in Fig D. The results from these nine approaches show that treatment-resistant depression patients had statistically significantly higher total cost to payers than non–treatment-resistance major depressive disorder patients.

**Table A. Additional 40 covariates selected vs HD1.**

| **Code** | **Code type** | **Description** | **Category** |
| --- | --- | --- | --- |
| V04.81 | ICD-9 | Need for prophylactic vaccination and inoculation against influenza | Respiratory |
| 780.79 | ICD-9 | Other malaise and fatigue | Fatigue |
| 272.4 | ICD-9 | Other and unspecified hyperlipidemia | Metabolic |
| 724.2 | ICD-9 | Lumbago | Pain |
| 729.5 | ICD-9 | Pain in limb | Pain |
| 789 | ICD-9 | Abdominal pain, unspecified site | Pain |
| 786.5 | ICD-9 | Chest pain, unspecified | Pain |
| 305.1 | ICD-9 | Tobacco use disorder | Tobacco/Rx |
| 530.81 | ICD-9 | Esophageal reflux | Metabolic |
| 784 | ICD-9 | Headache | Pain |
| 462 | ICD-9 | Acute pharyngitis | Metabolic |
| 599 | ICD-9 | Urinary tract infection, site not specified | Metabolic |
| 990 | ICD-9 | Effects of radiation, unspecified | Radiation |
| 723.1 | ICD-9 | Cervicalgia | Pain |
| 268.9 | ICD-9 | Unspecified vitamin D deficiency | Fatigue |
| 465.9 | ICD-9 | Acute upper respiratory infections of unspecified site | Respiratory |
| 786.2 | ICD-9 | Cough | Respiratory |
| 724.5 | ICD-9 | Backache, unspecified | Pain |
| 272 | ICD-9 | Pure hypercholesterolemia | Metabolic |
| 780.52 | ICD-9 | Insomnia, unspecified | Fatigue |
| 00173068220 | NDC | 200 ACTUAT Albuterol 0.09 MG/ACTUAT Metered Dose Inhaler [Ventolin] | Bronchodilator |
| 00406035705 | NDC | Acetaminophen 500 MG / Hydrocodone Bitartrate 5 MG Oral Tablet | Analgesic |
| 53746027205 | NDC | Sulfamethoxazole 800 MG / Trimethoprim 160 MG Oral Tablet | Antibiotics |
| 53746046605 | NDC | Ibuprofen 800 MG Oral Tablet | NSAIDs |
| 00172541211 | NDC | Fluconazole 150 MG Oral Tablet | Antifungal |
| 00591034905 | NDC | Acetaminophen 500 MG / Hydrocodone Bitartrate 5 MG Oral Tablet | Analgesic |
| 00781261305 | NDC | Amoxicillin 500 MG Oral Capsule | Antibiotics |
| 59746000103 | NDC | {21 (Methylprednisolone 4 MG Oral Tablet) } Pack | Corticosteroid |
| 16252051501 | NDC | Ciprofloxacin 500 MG Oral Tablet | Antibiotics |
| 00406051201 | NDC | Acetaminophen 325 MG / Oxycodone Hydrochloride 5 MG Oral Tablet | Analgesic |
| 50383070016 | NDC | 120 ACTUAT Fluticasone propionate 0.05 MG/ACTUAT Nasal Inhaler | Corticosteroid |
| 00591320201 | NDC | Acetaminophen 325 MG / Hydrocodone Bitartrate 5 MG Oral Tablet | Analgesic |
| 00054327099 | NDC | 120 ACTUAT Fluticasone propionate 0.05 MG/ACTUAT Nasal Inhaler | Corticosteroid |
| 00093314705 | NDC | Cephalexin 500 MG Oral Capsule | Antibiotics |
| 53746046505 | NDC | Ibuprofen 600 MG Oral Tablet | NSAIDs |
| 00093714618 | NDC | {6 (Azithromycin 250 MG Oral Tablet) } Pack | Antibiotics |
| 00591074905 | NDC | Acetaminophen 325 MG / Oxycodone Hydrochloride 5 MG Oral Tablet | Analgesic |
| 68382031910 | NDC | tramadol hydrochloride 50 MG Oral Tablet | Narcotic |
| 59746017710 | NDC | Cyclobenzaprine hydrochloride 10 MG Oral Tablet | Analgesic |
| 00781185220 | NDC | Amoxicillin 875 MG / Clavulanate 125 MG Oral Tablet | Antibiotics |

HD1, high dimensional covariate selection approach 1; ICD-9, *International Classification of Diseases, 9th Edition*; NDC, National Drug Code; NSAID, nonsteroidal anti-inflammatory drug.

**Table B. Additional 41 covariates selected vs HD2.**

| **Code** | **Code type** | **Description** | **Category** |
| --- | --- | --- | --- |
| 85025 | CPT/HCPCS | Blood count; complete (CBC), automated (Hgb, Hct, RBC, WBC, and platelet count) and automated differential WBC count | Test |
| 74177 | CPT/HCPCS | Computed tomography, abdomen and pelvis; | Diagnostic test |
| 82570 | CPT/HCPCS | Creatinine; other source | Test |
| 93010 | CPT/HCPCS | Electrocardiogram, routine ECG with at least 12 leads | ER visit |
| 99283 | CPT/HCPCS | Emergency department visit for the evaluation and management of a patient | ER visit |
| 99284 | CPT/HCPCS | Emergency department visit for the evaluation and management of a patient | ER visit |
| 99285 | CPT/HCPCS | Emergency department visit for the evaluation | ER visit |
| A0425 | CPT/HCPCS | Ground mileage, per statute mile | Exercise |
| 99223 | CPT/HCPCS | Initial hospital care, per day, for the evaluation and management of a patient | Inpatient visit |
| 88305 | CPT/HCPCS | Level IV - Surgical pathology, gross and microscopic examination is for the gross and microscopic examination of a specimen to provide a diagnosis | Exam |
| 99244 | CPT/HCPCS | Office consultation for a new or established patient | Outpatient visit |
| 99204 | CPT/HCPCS | Office or other outpatient visit for the evaluation and management of a new patient | Outpatient visit |
| 99205 | CPT/HCPCS | Office or other outpatient visit for the evaluation and management of a new patient | Outpatient visit |
| 99214 | CPT/HCPCS | Office or other outpatient visit for the evaluation and management of an established patient | Outpatient visit |
| 99213 | CPT/HCPCS | Office or other outpatient visit for the evaluation and management of an established patient | Outpatient visit |
| 71020 | CPT/HCPCS | Radiologic examination, chest, 2 views | Exam |
| 73630 | CPT/HCPCS | Radiologic examination, foot; complete, minimum of 3 views | Exam |
| 99232 | CPT/HCPCS | Subsequent hospital care, per day, for the evaluation and management of a patient | Inpatient visit |
| 76499 | CPT/HCPCS | Unlisted diagnostic radiographic procedure | Diagnostic test |
| 89240 | CPT/HCPCS | Unlisted miscellaneous pathology test | Diagnostic test |
| 81003 | CPT/HCPCS | Urinalysis, by dip stick or tablet reagent for bilirubin, glucose, hemoglobin, ketones, leukocytes, nitrite, pH, protein, specific gravity, urobilinogen, any number of these constituents | Test |
| 78907 | ICD-9 | Abdominal pain, generalized | Abdominal pain |
| 78909 | ICD-9 | Abdominal pain, other specified site | Abdominal pain |
| 78900 | ICD-9 | Abdominal pain, unspecified site | Abdominal pain |
| 78650 | ICD-9 | Chest pain, unspecified | Abdominal pain |
| 7862 | ICD-9 | Cough | Chest pain |
| 78791 | ICD-9 | Diarrhea | Other |
| 7820 | ICD-9 | Disturbance of skin sensation | GI problems |
| 7823 | ICD-9 | Edema | Other |
| 7840 | ICD-9 | Headache | Headache |
| V5869 | ICD-9 | Long-term (current) use of other medication | Long term medication |
| 7291 | ICD-9 | Myalgia and myositis, unspecified | GI problems |
| 78702 | ICD-9 | Nausea alone | GI problems |
| 78701 | ICD-9 | Nausea with vomiting | Other |
| 78079 | ICD-9 | Other malaise and fatigue | Other |
| 78609 | ICD-9 | Other respiratory abnormalities | Other |
| V7283 | ICD-9 | Other specified pre-operative examination | Exam |
| 71946 | ICD-9 | Pain in joint, lower leg | Other pain |
| 7295 | ICD-9 | Pain in limb | Other pain |
| 5990 | ICD-9 | Urinary tract infection, site not specified | Other |

HD2, high dimensional covariate selection approach 2; CPT, Current Procedural Terminology; HCPCS, Healthcare Common Procedure Coding System; CBC, complete blood count; RBC, red blood cell; WBC, white blood cell; ECG, electrocardiogram; ER, emergency room; ICD-9, *International Classification of Diseases, 9th Edition*; GI, gastrointestinal.

**Table C. Number of patients used in each analysis.**

| **Covariate set** | **Method** | **Treatment-resistant depression** | **Non–treatment-resistant major depressive disorder** |
| --- | --- | --- | --- |
| Pre-specified | PSM | 2370 | 9289 |
|  | MDM | 2384 | 9536 |
|  | PS_Strata | 2384 | 15475 |
| HD1 | PSM | 2366 | 9114 |
|  | MDM | 2384 | 9536 |
|  | PS_Strata | 2384 | 15475 |
| HD2 | PSM | 2356 | 9056 |
|  | MDM | 2384 | 9536 |
|  | PS_Strata | 2384 | 15475 |

PSM, propensity score matching; MDM, Mahalanobis distance matching; PS_Strata, stratification by propensity score; HD1, high dimensional covariate selection approach 1; HD2, high dimensional covariate selection approach 2.

**Fig D. Difference in least square means of** **total cost to payers (US$) estimate between treatment-resistant depression and non–treatment-resistant major depressive disorder.^a^**

^
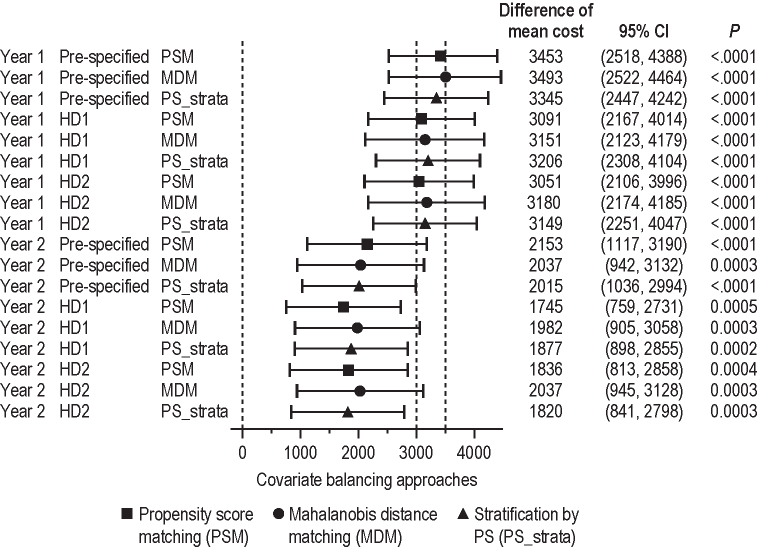
^

^a^The “Pre-specified, PSM” covariate set is the same one as used for the linear model approach in S4 Table.
