# Supplementary figures and images for: A retrospective analysis to estimate the healthcare resource utilization and cost associated with treatment-resistant depression in commercially insured US patients
Source: PLoS One. 2020 Sep 11;15(9):e0238843. doi: 10.1371/journal.pone.0238843 (PMC7485754; doi:10.1371/journal.pone.0238843)

**S1 Fig. Patient disposition.**


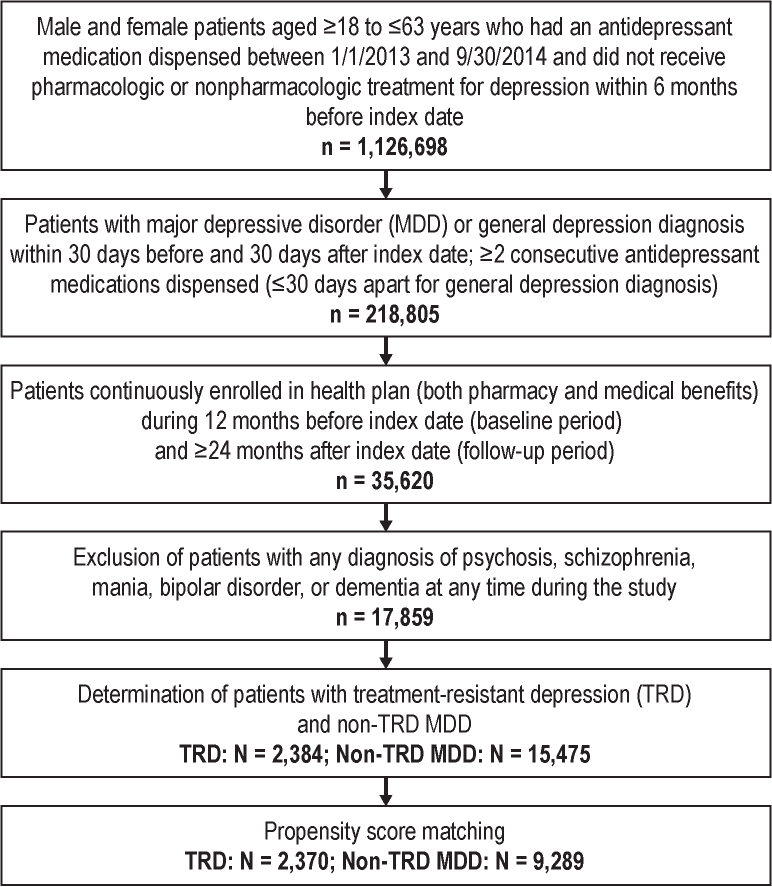

Supplement: S1 Fig — (DOCX) [file pone.0238843.s002.docx]
