# Supplementary material for: A retrospective analysis to estimate the healthcare resource utilization and cost associated with treatment-resistant depression in commercially insured US patients
Source: PLoS One. 2020 Sep 11;15(9):e0238843. doi: 10.1371/journal.pone.0238843 (PMC7485754; doi:10.1371/journal.pone.0238843)
Supplement: S1 Table — (DOCX) [file pone.0238843.s003.docx]

# S1 Table. List of antidepressant medications and minimum adequate dose.

| **Antidepressant medication** | **Minimum daily adequate dose^a^** |
| --- | --- |
| **SSRIs** | |
| Citalopram | 20 mg |
| Escitalopram | 10 mg |
| Fluvoxamine^b^ | 50 mg |
| Fluvoxamine, continuous release^b^ | 100 mg |
| Fluoxetine | 20 mg |
| Paroxetine | 20 mg |
| Paroxetine, extended release | 12.5 mg |
| Sertraline | 50 mg |
| Vilazodone^b^ | 10 mg |
| **DNRI** | |
| Bupropion | 150 mg |
| **SNRIs** | |
| Desvenlafaxine | 50 mg |
| Duloxetine | 60 mg |
| Levomilnacipran^b^ | 20 mg |
| Milnacipran^b^ | 12.5 mg |
| Venlafaxine | 37.5 mg |
| **Serotonin modulators** | |
| Nefazodone | 50 mg |
| Trazodone | 150 mg |
| Vortioxetine^b^ | 10 mg |
| **Tricyclics and tetracyclics** | |
| Amitriptyline | 25 mg |
| Amoxapine^b^ | 50 mg |
| Clomipramine^b^ | 25 mg |
| Doxepin | 25 mg |
| Desipramine | 25 mg |
| Imipramine | 25 mg |
| Maprotiline | 75 mg |
| Mirtazapine | 15 mg |
| Nortriptyline | 25 mg |
| Protriptyline | 10 mg |
| Trimipramine | 25 mg |
| **MAOIs** | |
| Isocarboxazid | 10 mg |
| Moclobemide | 150 mg |
| Phenelzine | 15 mg |
| Selegiline transdermal | 6 mg |
| Tranylcypromine | 10 mg |
| **Other selected medications^c^** | |
| Olanzapine-fluoxetine | 25 mg |
| Mianserin | NA^d^ |
| Nefazadone | NA^d^ |
| Agomelatine | NA^d^ |
| Tianeptine | NA^d^ |
| Reboxetine | NA^d^ |
| Opipramol | NA^d^ |
| Pipofezine | NA^d^ |
| Noxiptiline | NA^d^ |

MAOIs, monoamine oxidase inhibitors; NA, not applicable; NDRIs, norepinephrine-dopamine reuptake inhibitors; SNRIs, serotonin-norepinephrine reuptake inhibitors; SSRIs, selective serotonin reuptake inhibitors.

^a^Starting doses were based on the recommended starting dose indicated in the American Psychiatric Association (APA) Practice Guidelines for Treatment of Patients with Major Depressive Disorder, 3^rd^ edition, 2010 (https://psychiatryonline.org/pb/assets/raw/sitewide/practice_guidelines/guidelines/mdd.pdf).

^b^Starting doses for other antidepressant medications not included in the APA Practice Guidelines for Treatment of Patients with Major Depressive Disorder were based on the starting doses indicated in the label (<http://www.accessdata.fda.gov/scripts/cder/drugsatfda/index.cfm>).

^c^Other selected medications from the database include an antidepressant-antipsychotic combination treatment indicated for treatment resistant depression, a selected antianxiety agent, and other agents not approved for use in the United States (US Food and Drug Administration. Drugs@FDA: FDA-approved drugs. Available from: <https://www.accessdata.fda.gov/scripts/cder/daf/>).

^d^Not applicable; not approved for use in the United States.
