# Supplementary material for: A retrospective analysis to estimate the healthcare resource utilization and cost associated with treatment-resistant depression in commercially insured US patients
Source: PLoS One. 2020 Sep 11;15(9):e0238843. doi: 10.1371/journal.pone.0238843 (PMC7485754; doi:10.1371/journal.pone.0238843)
Supplement: S2 Table — (DOCX) [file pone.0238843.s004.docx]

**S2 Table. Comparison of costs per year (US$) between treatment-resistant depression and non–treatment-resistant major depressive disorder patients by the model with gamma log link vs a linear model.^a^**

|  | **Gamma Log Link** | | | | **Linear model** | | | |
| --- | --- | --- | --- | --- | --- | --- | --- | --- |
| **Variable** | **Treatment-resistant depression** | **Non–treatment-resistant major depressive disorder** | **Estimate of mean difference** | **95% CI** | **Treatment-resistant depression** | **Non–treatment-resistant major depressive disorder** | **Estimate of mean difference** | **95% CI** |
| Cost to payers | | | |  |  | | | |
| Medical cost in Year 1 | 9075 | 6125 | 2950 | (2051, 3978) | 9036 | 6111 | 2925 | (2047, 3802) |
| Medical cost in Year 2 | 8393 | 6621 | 1772 | (632, 2958) | 7813 | 6063 | 1750 | (767, 2733) |
| Pharmacy cost in Year 1 | 2043 | 1507 | 535 | (300, 789) | 1899 | 1377 | 522 | (298, 746) |
| Pharmacy cost in Year 2 | 2027 | 1664 | 362 | (58, 720) | 1724 | 1328 | 397 | (159, 634) |
| Total cost to payers in Year 1 | 11014 | 7585 | 3430 | (2438, 4478) | 10940 | 7487 | 3453 | (2518, 4388) |
| Total cost to payers in Year 2 | 10175 | 7984 | 2191 | (1031, 3453) | 9542 | 7389 | 2153 | (1117, 3190) |
| Cost to patients^b^ |  |  |  |  |  |  |  |  |
| Medical cost in Year 1 | 1373 | 1019 | 444 | (347, 556) | 1349 | 1009 | 340 | (260, 421) |
| Medical cost in Year 2 | 1207 | 1022 | 245 | (150, 344) | 1114 | 914 | 200 | (116, 284) |
| Prescription cost in Year 1 | 406 | 318 | 88 | (68, 109) | 393 | 316 | 77 | (58, 96) |
| Prescription cost in Year 2 | 350 | 301 | 49 | (30, 70) | 321 | 270 | 51 | (31, 71) |
| Total cost to patients in Year 1 | 1767 | 1323 | 354 | (260, 457) | 1743 | 1325 | 419 | (333, 504) |
| Total cost to patients in Year 2 | 1499 | 1254 | 184 | (91, 285) | 1436 | 1184 | 253 | (164, 341) |
| Total healthcare cost |  |  |  |  |  |  |  |  |
| Total healthcare cost in Year 1 | 12726 | 8881 | 3846 | (2855, 4928) | 12684 | 8811 | 3872 | (2896, 4848) |
| Total healthcare cost in Year 2 | 11591 | 9179 | 2412 | (1217, 3713) | 10979 | 8573 | 2407 | (1330, 3483) |

CI, confidence interval.

^a^Costs were calculated using a linear model with normal distribution in comparison to the model with gamma distribution with the log-link function.

^b^Cost to patients is the original data collection from patients. The amount was not adjusted based on inflation rate, geographic variation, or facility variation.

- **The results from the two models are consistent: treatment-resistant depression patients, compared with non–treatment-resistant major depressive disorder patients, had statistically significantly greater costs to payers and patients.**
