# Supplementary material for: A retrospective analysis to estimate the healthcare resource utilization and cost associated with treatment-resistant depression in commercially insured US patients
Source: PLoS One. 2020 Sep 11;15(9):e0238843. doi: 10.1371/journal.pone.0238843 (PMC7485754; doi:10.1371/journal.pone.0238843)
Supplement: S3 Table — (DOCX) [file pone.0238843.s005.docx]

**S3 Table. Difference in least square means of costs per year (US$) between treatment-resistant depression and non–treatment-resistant major depressive disorder patients by depression diagnosis codes (from linear models).^a^**

|  | **Major depressive disorder**  **(ICD-9 296.X)** | **Dysthymic disorder (ICD-9 300.X)** | **Adjustment disorder (ICD-9 309.X)** | **Depressive disorder NOS (ICD-9 311.X)** |
| --- | --- | --- | --- | --- |
| Number of patients | 4822 | 1557 | 270 | 5010 |
| Cost to payers, mean (95% CI) |  |  |  |  |
| Medical cost in Year 1 | 2863 (1502, 4224) | 2887 (1608, 4166) | 8762 (–4668, 22193) | 2602 (1285, 3919) |
| Medical cost in Year 2 | 885 (–707, 2478) | 1112 (–369, 2592) | 5427 (1836, 9019) | 2607 (975, 4238) |
| Pharmacy cost in Year 1 | 171 (–178, 521) | 496 (150, 841) | 796 (–997, 2590) | 853 (481, 1224) |
| Pharmacy cost in Year 2 | 267 (–47, 582) | 288 (–87, 662) | –435 (–2168, 1298) | 608 (168, 1048) |
| Total cost to payers in Year 1 | 3043 (1590, 4497) | 3380 (2024, 4736) | 9574 (–4420, 23569) | 3454 (2043, 4866) |
| Total cost to payers in Year 2 | 1162 (–506, 2830) | 1397 (–185, 2979) | 5009 (886, 9131) | 3214 (1489, 4940) |
| Cost to patients,^b^ mean (95% CI) |  |  |  |  |
| Medical cost in Year 1 | 270 (146, 394) | 290 (125, 456) | 669 (139, 1200) | 401 (268, 534) |
| Medical cost in Year 2 | 164 (10, 317) | 139 (–30, 307) | 667 (212, 1122) | 223 (110, 337) |
| Prescription cost in Year 1 | 86 (58, 114) | 61 (15, 107) | 42 (–71, 155) | 75 (44, 107) |
| Prescription cost in Year 2 | 64 (34, 94) | 65 (9, 121) | 25 (–87, 136) | 36 (5, 67) |
| Total cost to patients in Year 1 | 356 (226, 487) | 354 (177, 530) | 738 (182, 1295) | 477 (337, 618) |
| Total cost to patients in Year 2 | 228 (68, 388) | 206 (24, 388) | 719 (229, 1208) | 260 (139, 381) |
| Total healthcare cost, mean (95% CI) | |  |  |  |
| Total healthcare cost in Year 1 | 3400 (1891, 4909) | 3732 (2271, 5193) | 10283 (–3995, 24562) | 3933 (2444, 5421) |
| Total healthcare cost in Year 2 | 1390 (–340, 3120) | 1601 (–93, 3295) | 5697 (1214, 10181) | 3476 (1687, 5264) |

NOS, not otherwise specified; CI, confidence interval.

^a^Least square means were calculated from a linear model with normal distribution, adjusting for the baseline variable.

^b^Cost to patients is the original data collection from patients. The amount was not adjusted based on inflation rate, geographic variation, or facility variation.
