# Supplementary material for: A retrospective analysis to estimate the healthcare resource utilization and cost associated with treatment-resistant depression in commercially insured US patients
Source: PLoS One. 2020 Sep 11;15(9):e0238843. doi: 10.1371/journal.pone.0238843 (PMC7485754; doi:10.1371/journal.pone.0238843)
Supplement: S4 Table — (DOCX) [file pone.0238843.s006.docx]

**S4 Table. Demographic and clinical characteristics of patients with treatment-resistant depression and non–treatment-resistant depression before and after the propensity score matching at 1:1 ratio.**

| **Characteristic** | **Unmatched** | | | | | **Matched** | | | | |
| --- | --- | --- | --- | --- | --- | --- | --- | --- | --- | --- |
|  | **Treatment-resistant depression**  **(N = 2,384)** | | **Non–treatment-resistant major depressive disorder**  **(N = 15,475)** | |  | **Treatment-resistant depression**  **(n = 2,370)** | | **Non–treatment-resistant major depressive disorder**  **(n = 2,370)** | |  |
|  | **n** | **%** | **n** | **%** | ***P*** | **n** | **%** | **n** | **%** | ***P*** |
| Age (years) | Mean 39.2 | SD 13.0 | Mean 40.1 | SD 12.9 | 0.0021 | Mean 39.2 | SD 12.9 | Mean 38.9 | SD 12.8 | 0.4339 |
| Age group (years) |  |  |  |  |  |  |  |  |  |  |
| 18-24 | 472 | 20 | 2724 | 18 |  | 468 | 20 | 469 | 20 |  |
| 25-34 | 440 | 18 | 2779 | 18 |  | 437 | 18 | 445 | 19 |  |
| 35-44 | 585 | 25 | 3735 | 24 |  | 582 | 25 | 609 | 26 |  |
| 45-54 | 547 | 23 | 3657 | 24 |  | 545 | 23 | 524 | 22 |  |
| 55-63 | 340 | 14 | 2580 | 17 | 0.0082 | 338 | 14 | 323 | 14 | 0.8375 |
| Female sex | 1481 | 62 | 9209 | 60 | 0.0154 | 1474 | 62 | 1474 | 62 | 1.0000 |
| Comorbidities |  |  |  |  |  |  |  |  |  |  |
| Anxiety | 659 | 28 | 3664 | 24 | <.0001 | 646 | 27 | 649 | 27 | 0.9221 |
| Personality Disorder | 17 | 1 | 53 | <1 | 0.0813 | 8 | <1 | 4 | <1 | 0.2476 |
| Substance Abuse | 73 | 3 | 392 | 3 | 0.1056 | 71 | 3 | 43 | 2 | 0.0079 |
| PTSD | 37 | 2 | 179 | 1 | <.0001 | 35 | 2 | 26 | 1 | 0.2461 |
| Elixhauser score^a^ | Mean 1.8 | SD 1.35 | Mean 1.70 | SD 1.26 | 0.0645 | Mean 1.74 | SD 1.33 | Mean 1.67 | SD 1.12 | 0.049 |
| Major depressive disorder diagnostic code |  |  |  |  |  |  |  |  |  |  |
| Major depressive disorder (ICD-9 296.X) | 1003 | 42 | 5485 | 35 |  | 991 | 42 | 1013 | 43 |  |
| Dysthymic disorder (ICD-9 300.X) | 323 | 14 | 2307 | 15 |  | 322 | 14 | 308 | 13 |  |
| Adjustment disorder (ICD-9 309.X) | 65 | 3 | 520 | 3 |  | 65 | 3 | 47 | 2 |  |
| Depressive disorder NOS (ICD-9 311.X) | 993 | 42 | 7163 | 46 | <.0001 | 992 | 42 | 1002 | 42 | 0.3213 |

CI, confidence interval; SD, standard deviation.

^a^The Elixhauser score ranges from 0 to 30.
