# Supplementary material for: A retrospective analysis to estimate the healthcare resource utilization and cost associated with treatment-resistant depression in commercially insured US patients
Source: PLoS One. 2020 Sep 11;15(9):e0238843. doi: 10.1371/journal.pone.0238843 (PMC7485754; doi:10.1371/journal.pone.0238843)
Supplement: S5 Table — (DOCX) [file pone.0238843.s007.docx]

**S5 Table. Healthcare resource utilization per year during the study period (matched at 1:1 ratio).**

| **Variable** | **Treatment-resistant depression** | | **Non–treatment-resistant major depressive disorder** | | **Treatment-resistant depression vs non–treatment-resistant major depressive disorder** | | |
| --- | --- | --- | --- | --- | --- | --- | --- |
|  | **n** | **%** | **n** | **%** | **Odds ratio** | **95% CI** | |
| Patients with ED visits in Year 1, % | 525 | 22 | 425 | 18 | 1.27 | 1.10 | 1.47 |
| Patients with ED visits in Year 2, % | 462 | 20 | 388 | 16 | 1.20 | 1.03 | 1.40 |
| Patients with inpatient hospitalization in Year 1, % | 207 | 9 | 142 | 6 | 1.45 | 1.16 | 1.81 |
| Patients with inpatient hospitalization in Year 2, % | 172 | 7 | 115 | 5 | 1.47 | 1.15 | 1.89 |
| **Variable** | **Treatment-resistant depression** | | **Non–treatment-resistant major depressive disorder** | | **Treatment-resistant depression vs non–treatment-resistant major depressive disorder** | | |
|  | **n** | | **n** | | **Estimate of mean difference** | **95% CI** | |
| Hospital LOS in Year 1, number of days | 8.22 | | 6.51 | | 1.71 | -1.15 | 4.57 |
| Hospital LOS in Year 2, number of days | 9.27 | | 6.48 | | 2.79 | -4.40 | 9.98 |
| Number of outpatient visits in Year 1 | 11.3 | | 8.65 | | 2.65 | 2.14 | 3.15 |
| Number of outpatient visits in Year 2 | 7.22 | | 5.64 | | 1.38 | 0.91 | 1.84 |

CI, confidence interval; ED, emergency department; LOS, length of stay.
