# Supplementary material for: A retrospective analysis to estimate the healthcare resource utilization and cost associated with treatment-resistant depression in commercially insured US patients
Source: PLoS One. 2020 Sep 11;15(9):e0238843. doi: 10.1371/journal.pone.0238843 (PMC7485754; doi:10.1371/journal.pone.0238843)
Supplement: S6 Table — (DOCX) [file pone.0238843.s008.docx]

**S6 Table. Comparison of costs per year between treatment-resistant depression and non–treatment-resistant major depressive disorder patients (US$; matched at 1:1 ratio).^a^**

| **Variable** | **Treatment-resistant depression** | **Non–treatment-resistant major depressive disorder** | **Treatment-resistant depression vs non–treatment-resistant major depressive disorder** | | |
| --- | --- | --- | --- | --- | --- |
|  |  |  | **Adjusted mean difference** | **95% CI** | |
| **Cost to payers** | | | | | |
| Medical cost in Year 1 | 8908 | 6027 | 2881 | 1913 | 3909 |
| Medical cost in Year 2 | 8276 | 6296 | 1980 | 843.4 | 3226 |
| Pharmacy cost in Year 1 | 2025 | 1425 | 600.2 | 308.4 | 886.5 |
| Pharmacy cost in Year 2 | 1905 | 1493 | 411.5 | 34.3 | 819.2 |
| Total cost to payers in Year 1 | 10831 | 7420 | 3411 | 2433 | 4496 |
| Total cost to payers in Year 2 | 9963 | 7544 | 2419 | 1232 | 3766 |
| **Cost to patients** | | | | | |
| Medical cost in Year 1 | 1371 | 995 | 375 | 273 | 485 |
| Medical cost in Year 2 | 1208 | 1003 | 206 | 101 | 313 |
| Prescription cost in Year 1 | 406 | 318 | 88 | 63 | 111 |
| Prescription cost in Year 2 | 350 | 295 | 55 | 30 | 79 |
| Total cost to patients in Year 1 | 1766 | 1299 | 467 | 361 | 573 |
| Total cost to patients in Year 2 | 1500 | 1234 | 267 | 153 | 377 |
| **Total healthcare cost** | | | | | |
| Total healthcare cost in Year 1 | 12586 | 8714 | 3872 | 2829 | 5036 |
| Total healthcare cost in Year 2 | 11422 | 8755 | 2668 | 1466 | 4014 |

CI, confidence interval.

^a^Medical costs to payers included claims for outpatient visits, ED visits, and hospitalizations; pharmacy costs to payers were the sum of pharmacy claims; and total costs to payers were the sum of medical costs and pharmacy costs to payers. Medical costs to patients were defined as the sum of deductibles, copayments, and coinsurance for all medical services; prescription costs to patients were defined as the sum of deductibles, copayments, and coinsurance for all prescription drugs; and total costs to patients were the sum of medical costs and prescription costs to patients. Total healthcare costs were defined as the sum of costs to payers and patients.
